# Effect of hydro-oleophobic perfluorocarbon chain on interfacial behavior and mechanism of perfluorooctane sulfonate in oil-water mixture

**DOI:** 10.1038/srep44694

**Published:** 2017-03-16

**Authors:** Pingping Meng, Shubo Deng, Ziwen Du, Bin Wang, Jun Huang, Yujue Wang, Gang Yu, Baoshan Xing

**Affiliations:** 1 State Key Joint Laboratory of Environment Simulation and Pollution Control, School of Environment, POPs Research Center, Tsinghua University, Beijing, 100084, China; 2 Stockbridge School of Agriculture, University of Massachusetts, Amherst, Massachusetts, 01003, USA

## Abstract

Perfluorocarbon chain of perfluorooctane sulfonate (PFOS) is not only hydrophobic but also oleophobic, and its effect on PFOS distribution in oil-water mixture and underlying mechanism are unclear. For the first time, we propose that PFOS can emulsify oil-water mixture only in the presence of air, completely different from hydrocarbon surfactants. The perfluorocarbon chain repels hydrophobic compounds and its oleophobicity increases with decreasing polarity of organic solvents. The formed emulsion in oil phase contains high concentrations of PFOS, resulting in PFOS decrease in water. The increase of shaking speed and time as well as oil and air volume all increase the emulsification and decrease PFOS concentrations in water. During the settling process, the emulsion gradually disappears and the concentrated PFOS is released into water. The emulsification mechanism of PFOS based on air bubbles is proposed, and PFOS partitions to the interfaces of air bubbles with the hydro-oleophobic perfluorocarbon chain stretching into air bubbles and the polar head in water. This study clarifies the ambiguous understanding of the oleophobicity of perfluorocarbon chain in PFOS, and it is helpful for the understanding of the transport and fate of PFOS at oil-water interfaces in aquatic environments as well as the enhanced removal of PFOS from wastewater.

Perfluorinated compounds (PFCs) have drawn great attention in recent years^1^,^2^,^3^,^4^. Perfluorooctane sulfonate (PFOS) is one of the typical PFCs, and has been widely produced and used in many industries^5^,^6^,^7^. PFOS occurrence in aquatic environments is a long-term environmental problem. PFOS has unique physicochemical and biological properties^5^,^6^, which may directly affect its transport and fate in aquatic environments as well as their removal efficiency from water. Conventional organic pollutants tend to accumulate in fat tissues of human bodies or animals, while PFOS is prone to accumulate in kidney, liver and serum^7^,^8^,^9^. It is the oleophobic perfluorocarbon chain that drives PFOS to stay away from fat tissues, while sulfonic group of PFOS may interact with the positively charged amino group in kidney or serum^6^,^10^,^11^,^12^. Furthermore, the oleophobicity of perfluorocarbon chain could be manifested by extremely large contact angles of oils on fluorinated surfaces^13^, and some superhydrophobic and self-cleaning coatings or membranes have been developed via the decoration of fluoroalkyl chain on the surface^14^,^15^,^16^,^17^,^18^.

However, some researchers reported that PFOS exhibited the same performance as conventional organic pollutants, regardless of the oleophobicity of perfluorocarbon chain. When activated carbon and carbon nanotubes were used to remove PFOS, hydrophobic interaction between the adsorbents and perfluorocarbon chain of PFOS was regarded as the main adsorption mechanism^19^,^20^,^21^, similar to conventional organic compounds. Also, it was observed that PFCs enrichment in sediment increased with increasing organic matter content, attributed to their hydrophobic interaction^22^,^23^. Recently, the distribution of different perfluoroalkyl acids (PFAAs) in soil-trichloroethylene-water system with the involvement of sodium dodecyl sulfate (SDS) has been studied^24^, and the joint formation of hemi-micelles by mixed SDS and PFAAs (the attachment of perfluorocarbon chain on hydrocarbon chain) was attributed to the enhanced sorption of PFAAs on soils. These phenomena are not consistent with the oleophobicity of perfluorocarbon chain, which stays away from hydrophobic oil phases.

Evidently, the understanding of the hydro-oleophobicity of perfluorocarbon chain at environmental interfaces is currently ambiguous, therefore further study is required to clarify the role of hydro-oleophobicity in the environmental processes of PFOS. Actually, PFOS preferably partitions to the air-water interfaces, rather than water-oil interfaces^25^. Fluorinated surfactants have been found to concentrate at the CO_2_-water interfaces, and significantly decrease the interfacial tension^26^,^27^,^28^. Aqueous film forming foam (AFFF, a kind of fire extinguishing agent) with high concentration of PFOS is often used to extinguish oil fire, and the produced wastewater contains lot of oils and air bubbles, but the distribution of PFOS at the interfaces in oily wastewater has not been reported. Our previous study showed that nano-air bubbles on hydrophobic carbonaceous adsorbents were responsible for PFOS adsorption, rather than the hydrophobic interaction^29^. Therefore, we assume that air bubbles might also be involved in PFOS fate in oil-water systems based on its hydro-oleophobicity, which has also never been addressed before.

In this study, typical oil-water mixtures are selected to investigate the transport and distribution of PFOS, especially the essential role of air bubbles in the system is examined. Hexane and octanol are selected as oils with different polarity, and PFOS and octyl sulfonate (OS) are used as the representative perfluorocarbon and hydrocarbon surfactants, respectively. Hexane is a widely used organic solvent, while octanol is similar to the carbohydrates and fat acid in human bodies and often used to simulate the biological system^30^,^31^. The emulsification of oil and PFOS solution in the presence/absence of air is compared, and PFOS distribution in the mixture as well as the hydro-oleophobicity of perfluorocarbon chain of PFOS are also studied. Finally, the interfacial mechanism of PFOS distribution in oil-water mixtures invoking air bubbles is proposed.

## Results and Discussion

**Figure 1:**
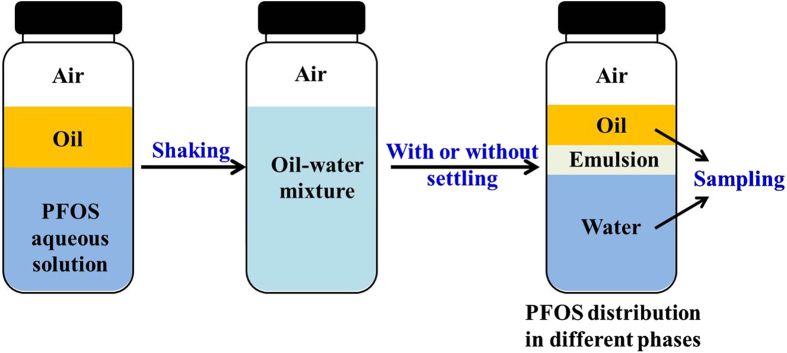
Schematic diagram for the experiments of PFOS distribution in the different phases of oil-water mixture.

Bottle tests were used to investigate the distribution of PFOS in oil-water mixture (Fig. 1). PFOS aqueous solution and oil were shaken in a bottle for emulsification, and samples were taken from the water phase or both the water and oil phases to measure PFOS concentrations after settling for different time. PFOS distribution in the emulsion was calculated from the difference between the total PFOS added and PFOS in water and oil phases. To rule out the effect of air bubbles, the bottles were filled with PFOS solution without space left. In the investigation of the effects of shaking conditions and air bubbles on PFOS concentrations in water, the clear water phase was available after shaking process, and samples were taken from the water phase without settling process.

We concern about the transport and distribution of PFOS during the mixing and settling processes. Besides water, oil and air phases, there are oil-water interface, air-water interface, and oil-air interface in the system. Due to the hydro-oleophobic perfluorocarbon chain of PFOS, we assume that PFOS prefers to stay at the air-water interface in the emulsion, rather than at oil-water interface or in the bulk oil phase.

### Effect of Shaking Conditions on PFOS Concentrations in Water

**Figure 2:**
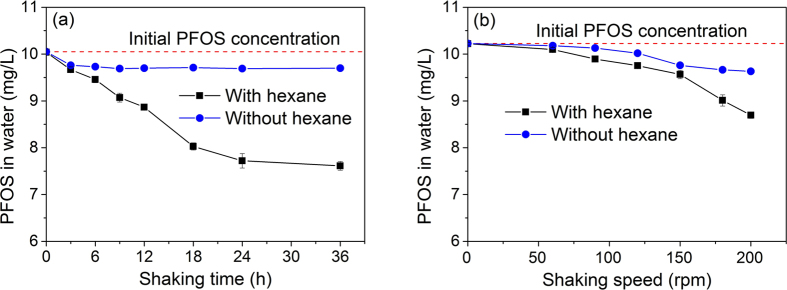
Effects of shaking time (shaking speed of 150 rpm) (**a**) and shaking speed (shaking time of 3 h) (**b**) on PFOS concentrations in water phase with the presence or absence of hexane after the vials were shaken for 24 h (0 min settling time).

During the fire extinguishing process, a large volume of AFFF solution is violently mixed with the burning oil. To simulate this process, the mixture of hexane and PFOS solution were shaken under different conditions. The initial PFOS concentration in aqueous solution is nearly 10 mg/L. Shaking speed and time influence the emulsification and distribution of PFOS in the mixture, and the concentrations of PFOS in water under different shaking conditions are shown in Fig. 2. With the increase of shaking time, PFOS concentrations in water decrease linearly within 24 h and then decrease slowly (Fig. 2a). About 76% of the initial PFOS concentration remains in water after 36 h shaking. PFOS concentrations in water also decrease with the increase of shaking speed, and the decrease of PFOS concentrations becomes more obvious at higher shaking speeds (Fig. 2b). The control experiment without hexane shows that the foam formed on water surface also causes the decrease of PFOS concentrations in water, but the decrease percent is much lower. An emulsion layer in hexane phase is observed, and its thickness increases with the increase of shaking time and speed (Figures S1 and S2). Almost the whole volume of hexane is emulsified after 12 h shaking. The emulsion in hexane becomes compact after long time and strong shaking, and the water phase gradually becomes turbid. Since the concentration of PFOS in clear hexane is measured to be about 12 μg/L, the amount of decreased PFOS in water should be concentrated in the emulsion. These results imply special environmental processes and potential remediation technology. Both oils and PFOS coexist in surface water or wastewater, natural and manmade stirring would alter the distribution of PFOS in water and oils. For surface water or seawater contaminated by oils, perfluorinated surfactants such as PFOS in water may gradually transfer into oil phase under the natural conditions, altering PFOS distribution in aquatic environments. If real wastewater contains high concentration of PFOS and a large amount of oil, strong agitation should be favorable for PFOS transport from water to oil phase.

### Effect of Air on PFOS Distribution in Oil-Water Mixture

**Figure 3:**
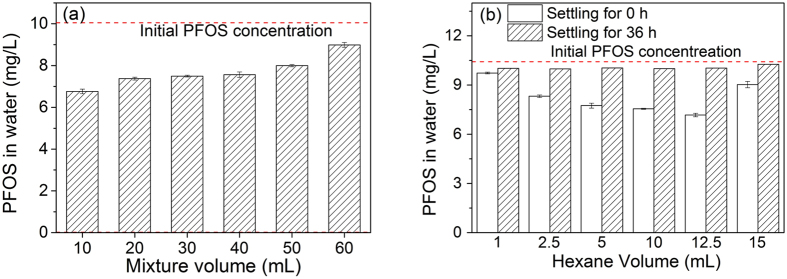
Effect of mixture volume on PFOS concentrations in water after shaking hexane-water (1:3 v/v) mixture at 150 rpm for 24 h in 60-mL vials (**a**), as well as effect of hexane volume on PFOS concentrations in 15 mL water after shaking the hexane-water mixture at 150 rpm for 24 h in a 30-mL vials and settling for 0 or 36 h (**b**).

Figure 3a presents the effect of mixture volume (or air volume) on PFOS concentrations in water after shaking the mixture of hexane and PFOS solution. When the mixture volume is increased from 10 to 50 mL in 60-mL vials, PFOS concentrations in water gradually increase from 6.8 to 8.0 mg/L, lower than the initial PFOS concentration of 10 mg/L. The mixture containing 7.5 mL of 10 mg/L PFOS solution and 2.5 mL of hexane in 60-mL vial was shaken for 24 h, following by removing the solvents using a nitrogen evaporator, and finally water was added to reach the initial 7.5 mL. The PFOS concentration was measured to be 10 mg/L, indicating that no PFOS was evaporated into the air phase in the vial. The adsorption of PFOS on the vials is found to be negligible via measuring the concentration difference of PFOS solution before and after adsorption. When the vial is filled with 60 mL mixture (remaining 2 mL airspace), PFOS concentration is 9.0 mg/L after shaking, and only a little emulsion is observed in hexane, unlike the full emulsion in hexane in other vials (Figure S3). These results indicate that both air and mixing strength may influence the emulsion formation. In consideration of less vigorous mixing in the large volume of mixture in the vials by shaking, different volumes of mixtures were added in the flasks and stirred using a magnetic stirrer. Although the mixing strength with a magnetic stirrer is different from that with shaking, it maintains the same stirring strength in these flasks with different volumes of solution. It is clear that the concentrations of PFOS in water increase with increasing volume of mixture (Figure S4), suggesting that air plays an important role in the emulsion formation, thus affecting the residual concentrations of PFOS in water.

Since the emulsion exists in hexane, the volume of hexane may affect PFOS distribution. With hexane volume increasing from 1 mL to 10 mL (constant volume of PFOS solution), PFOS concentrations in water after shaking decrease from 9.7 to 7.2 mg/L (Fig. 3b), indicating the important role of hexane volume in emulsion formation and PFOS concentrations in water. The contribution of PFOS dissolution in the increased volume of hexane is negligible due to its low solubility in hexane, and the control experimental result also rules out the influence of PFOS dissolution in the increased volume of hexane (Fig. 3b). Hexane volume limits the volume of the formed emulsion. Since some emulsion is present in water (turbid in Figure S5), the real residual PFOS concentrations in water should be lower than the values obtained. When 15 mL of hexane and 15 mL of PFOS solution are added in a 30-mL vial (with 2 mL airspace), the residual PFOS concentration after shaking is 9.0 mg/L and a little emulsion is formed (Figs 3b and S5>), further telling the important role of air in the formation of emulsion. Evidently, both air and hexane volume strongly affect PFOS concentrations in water, and higher volumes of air and hexane transfer more PFOS into hexane.

**Figure 4:**
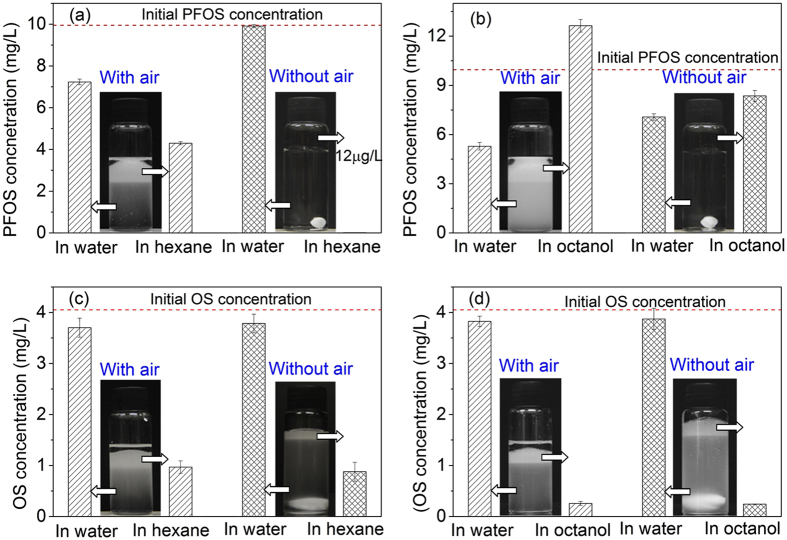
PFOS and OS distributions in hexane-water mixture after 0 min settling (**a**,**c**) and octanol-water mixture after 10 min settling (**b**,**d**) in the presence (space left in vials) or absence of air (completely full of mixture in the vials).

To further investigate the effect of air on PFOS distribution in the oil-water mixture, hexane and octanol are selected to represent nonpolar and polar oils, and PFOS distribution in water and oils after shaking their mixtures in the presence/absence of air is compared. In consideration of different mixing with and without air in the vials, a magnetic stirrer is added to aid mixing in the vial without air. Since the emulsion formed in the presence of octyl sulfonate using the magnetic stirrer in a vial without air is very similar to that obtained by shaking in the vial with air, the mixing strength in two cases should be similar. No emulsion is formed after shaking when the vial is completely filled with hexane-water mixture (Fig. 4a). The PFOS concentration in water is measured to be 9.9 mg/L, very close to its initial concentration of 10 mg/L, and only 12 μg/L of PFOS concentration is detected in hexane. By contrast, 5 mL of hexane and 15 mL of 10 mg/L PFOS solution are added in a 30-mL vial, PFOS concentrations in water and hexane after shaking are 7.3 and 4.2 mg/L, respectively. The emulsion mainly exists in hexane and a little emulsion is suspended in water, and the foam is visible in the emulsion. The emulsion in hexane is observed by a microscope, and many water droplets in the size range of 1–122 μm are present in the continuous hexane phase, but air bubbles could not be distinguished from water droplets (Figures S6 and S7). Thus, air facilitates the formation of emulsion and some PFOS molecules are transferred to hexane phase. In the octanol-water system, PFOS concentrations are measured after 10 min settling when a clear octanol layer is observed. Since octanol-water mixture in the presence of air was mixed uniformly after 24 h shaking and no clear octanol and water phases are observed, it is impossible to measure PFOS distribution in octanol and water phases without settling. PFOS concentrations in water and octanol after 10 min settling are 6.9 and 8.6 mg/L, respectively, if no airspace is left in the vial, while they change to 5.5 and 12.4 mg/L when 10 mL space is remained (Fig. 4b). Since air bubbles are ubiquitous in water and wastewater, they would significantly affect the transport and fate of PFOS in aquatic environment as well as PFOS removal from wastewater. The continuous lapping waves on ocean surfaces produce a large quantity of foam, making PFOS to be concentrated on sea surfaces^32^. PFOS adsorption on activated sludge in wastewater treatment may be related to PFOS accumulation at the surface of air bubbles attached on activated sludge during aeration process^33^.

To distinguish different types of emulsification between PFOS and hydrocarbon surfactants in oil-water mixture, octyl sulfonate (OS) distribution in hexane-water and octanol-water mixtures after shaking in the presence or absence of air was investigated. Both mixtures are emulsified no matter with or without air (Fig. 4c,d). In hexane-water mixture, OS concentrations in water are 3.70 mg/L in the presence of air and 3.78 mg/L in the case of no air, and OS concentrations in hexane are 0.97 and 0.88 mg/L, respectively. Similarly, OS concentrations in octanol and water in the presence of air are almost the same as the values in the absence of air. These results indicate that air has no effect on OS distribution in the mixture, completely different from PFOS. Hydrocarbon surfactants like OS could emulsify the hexane-water mixture by partitioning to the water-hexane interfaces, with its sulfonate group staying in water and hydrocarbon chain stretching into hexane phase.

The effect of initial PFOS concentrations on PFOS distribution in water and hexane/octanol is also studied (Figure S8). In the hexane-water mixture, almost all PFOS are present in water in case of no air, while PFOS concentrations in hexane increase from 2.7 to 46.8 mg/L in the presence of air when the initial PFOS concentrations increase from 5 to 100 mg/L. Similarly, the presence of air in the vials significantly increases the PFOS concentrations in octanol and decreases the PFOS concentrations in water. These results imply that PFOS mainly partitions to the gas-liquid interfaces in the presence of air.

### Emulsion Stability and PFOS Distribution in Settling Process

When PFOS solution without any oil was shaken in a vial, a foam layer is observed at the solution surface, and PFOS concentrations in solution also decrease from 10.5 to 9.2 mg/L (Figure S9). With the increase of settling time, PFOS concentrations in solution increase fast within 5 min, and almost recover the initial concentration at 10 min, attributed to the disappearance of foam (Figure S10). Hence, the foam formed by PFOS at the solution surface is not stable and disappears fast.

**Figure 5:**
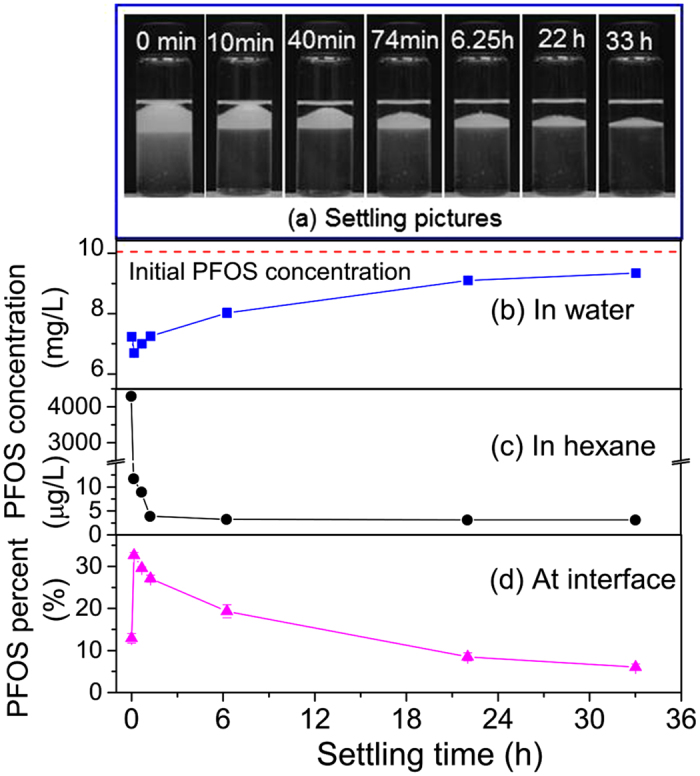
Settling photos of hexane-water mixture (**a**) as well as PFOS distribution in water (**b**), hexane (**c**), and at interfaces of air bubbles during the settling process.

In hexane-water and octanol-water mixtures, the emulsion stability is observed and PFOS concentrations in water and hexane/octanol phases are measured at different settling time. Figure 5 shows the emulsion change as well as PFOS distribution in water, hexane, and at the interfaces of air bubbles when the mixture of hexane and PFOS solution is shaken and then settled for different periods of time. After shaking, the upper hexane phase is filled with emulsion, and water phase is a little turbid (Fig. 5a). With increasing settling time, water becomes clear and emulsion becomes thinner and thinner at the bottom of hexane phase, indicating the disappearance of emulsion. After shaking, PFOS concentration in water is measured to be 7.3 mg/L, and it decreases to 6.6 mg/L after 10 min settling (Fig. 5b), due to the decrease of suspended emulsion in water. The PFOS concentrations in water increase gradually with increasing time from 10 min to 33 h, suggesting that some PFOS molecules are released into water when the emulsion disappears. In hexane, PFOS concentration is up to 4.2 mg/L at the beginning of settling, but it decreases fast to 12.2 μg/L after 10 min settling, and then keeps stable at 3 μg/L after 74 min settling (Fig. 5c). The amounts of PFOS at the interfaces of air bubbles are calculated by the total PFOS minus PFOS in water and hexane phases. The decrease of PFOS percent at interfaces with increasing settling time is observed from 33.5% at 10 min to 6.1% at 33 h (Fig. 5d), indicating that some air bubbles disappear and the interfacial PFOS molecules are released into water phase again. The low percent of PFOS at interface at 0 min settling is due to suspended emulsion in hexane and water after shaking, which causes the overestimated concentrations of PFOS in water and hexane phases. Since PFOS does not partition strongly to hexane, the released PFOS only enters water phase. The extra high PFOS concentration in hexane after shaking is attributed to the presence of air bubbles in the emulsion.

**Figure 6:**
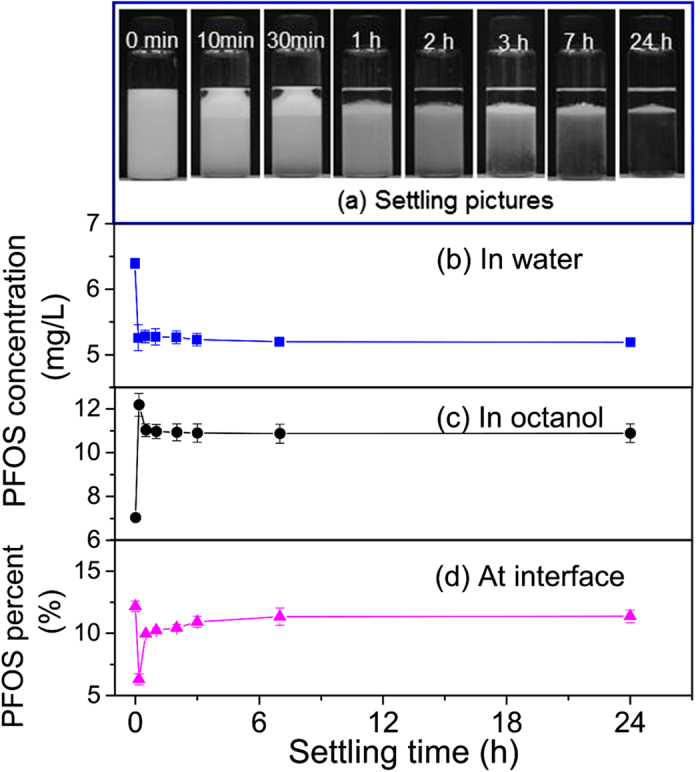
Settling photos of octanol-water mixture (**a**) as well as PFOS distribution in water (**b**), octanol (**c**), and at interfaces of air bubbles (**d**) during the settling process.

Figure 6 presents the settling process of octanol-water mixture as well as PFOS distribution in water, octanol, and at the interfaces of air bubbles. The mixture is completely emulsified after shaking (0 min settling, Fig. 6a). Since octanol is more hydrophilic than hexane, making it more soluble in water. In contrast with hexane, octanol is more easily emulsified with water in the presence of PFOS. During the settling process, the suspended emulsion in water goes up slowly, while the upper octanol phase becomes transparent within 1 h. The water phase is transparent from bottom after 2 h and is completely transparent at 24 h. The white emulsion is located at the bottom of the octanol phase. The formed emulsion in octanol-water mixture is much more stable than that in hexane-water mixture. Also, since PFOS is more soluble in octanol than in hexane, PFOS may dissolve in octanol instead of being immediately released back into the water phase when the emulsion disappears. PFOS concentrations in water decrease fast at the beginning of 10 min, and then decrease slowly from 5.4 mg/L at 10 min to 5.2 mg/L at 24 h (Fig. 6b), much lower than initial concentration of 10 mg/L. By contrast, PFOS concentrations in octanol increase significantly within 10 min settling and then decrease slowly from 11.5 mg/L at 10 min to 10.6 mg/L at 3 h (Fig. 6c), suggesting that PFOS is concentrated in octanol very fast. Thus, the significant change of PFOS concentrations in water and octanol at the beginning is due to the well-dispersed emulsion in the mixture. With the increase of settling time, PFOS concentrations in water and octanol all decrease gradually because more emulsions move to the bottom of octanol phase. The slow change of PFOS concentrations in water may be balanced by the rising emulsion (PFOS decrease) and emulsion disappearance (PFOS increase). After 7 h, the emulsion at the interface of octanol and water becomes stable, and about 12.8% of total PFOS is concentrated in the emulsion (Fig. 6d). It should be pointed out that the calculated percents of PFOS at interfaces of air bubbles before 7 h are not reliable because a lot of suspended emulsion is present in water phase.

In consideration of different PFOS concentrations in actual surface water or wastewater^34^,^35^,^36^, the effect of initial PFOS concentrations on PFOS distribution in hexane-water mixture is investigated. Since PFOS concentrations in actual surface water or wastewater are usually below 1 mg/L, the variable PFOS concentrations from 10 μg/L to 1 mg/L are selected to investigate the effect of initial PFOS concentrations on PFOS distribution in hexane-water mixture. When initial PFOS concentrations increased from 10 μg/L to 1 mg/L, the residual PFOS concentrations in water after shaking were from 1.3 μg/L to 549.3 μg/L (Figure S11). These results indicate that higher percent of PFOS is transferred to hexane when the initial PFOS concentration in water is lower. Even the concentration of PFOS is 10 μg/L, the emulsion can be observed in hexane phase after shaking, and more emulsion is formed at higher PFOS concentrations (Figure S12). After 1 h settling, only a little emulsion remained at the interface of hexane and water. It has been reported that some wastewaters contain high concentration of PFOS at mg/L level, and the concentrations of PFOS in surface water are in the level of μg/L and ng/L^20^,^34^,^37^. PFOS at high concentrations in water or wastewater would partition to the interfaces of air bubbles in organic phase if oils are present in the system and air may be involved in the formation of emulsion with the help of the external force.

### Hydrophobicity and Oleophobicity of Perfluorocarbon Chain in PFOS

**Figure 7:**
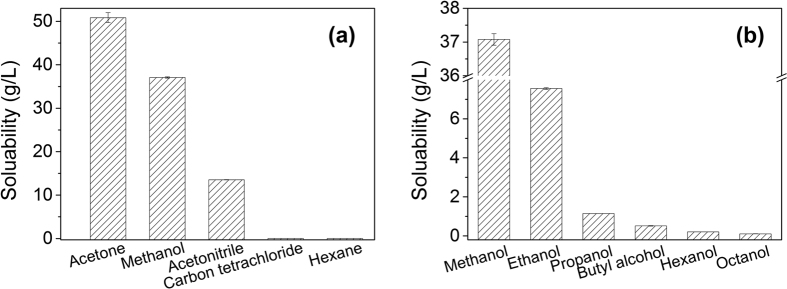
PFOS solubility in different organic solvents (**a**) and alcoholic solvents (**b**).

The oleophobicity of the perfluorocarbon chain affects the different behavior of PFOS in oil-water mixture, but the understanding of the oleophobic perfluorocarbon chain is still ambiguous. PFOS solubility in different organic solvents is presented in Fig. 7. According to the dielectric constants of organic solvents, the polarity of organic solvents decreased in the order of methanol > acetonitrile > acetone > carbon tetrachloride > hexane. Except for acetone, the solubility of PFOS in other solvents decreases with their decreasing polarity (Fig. 7a). Since PFOS contains a polar sulfonic acid group, it is very soluble in the highly polar solvents. PFOS solubility in acetone is higher than that in methanol because the cavity energy for acetone is smaller than that for methanol^38^. PFOS solubility values in the nonpolar solvents including carbon tetrachloride and hexane are 25 and 18 mg/L, respectively, much lower than those in polar methanol, acetonitrile, and acetone. Carbon tetrachloride has a lower equivalent alkane carbon number (EACN, assigned to an alkane carbon number implying equivalent alkane characteristics) than hexane, and surfactants have higher propensity to partition into a lower EACN oils^39^,^40^. It is notable that PFOS concentrations in hexane and octanol are up to 46.8 and 233.0 mg/L, respectively (Figure S8), when the initial PFOS concentration is 100 mg/L, much higher than their solubility obtained in Fig. 7. These results are consistent with that PFOS mainly partitions to the gas-liquid interfaces in the presence of air^25^, which is also seen in Figs 5d and 6d. In this study, the gas-liquid interfaces refer to the gas bubbles in emulsion. Figure 7b shows the solubility of PFOS in different alcoholic solvents, and PFOS solubility values decrease with increasing hydrocarbon chain length in the solvents. The solubility of PFOS in methanol is up to 37.1 g/L, while this value decreases to 0.11 g/L in octanol. The lower solubility of PFOS in lower polar solvents is contrary to that of hydrocarbon compounds^41^, indicating that the perfluorocarbon chain of PFOS is oleophobic and repels the hydrophobic parts of organic solvents.

To further verify the hydro-oleophobicity of perfluorocarbon chain in PFOS, perfluorooctane is mixed with the same volume of water or hexane since it has the same perfluorocarbon chain as PFOS. After shaking, the mixtures are settled for different time. The emulsion at the interfaces of water-perfluorooctane and hexane-perfluorooctane mixtures is observed after shaking, and the hexane-perfluorooctane mixture has more obvious emulsified layer than the water-perfluorooctane mixture (Figure S13a and b), suggesting that perfluorooctane has a higher repulsive force for water than for hexane, i.e., hydrophobicity of perfluorooctane is greater than its oleophobicity for hexane. Both emulsified layers disappear fast and the mixtures become transparent within 3 min. When hexane, water and perfluorooctane are mixed together, they separate quickly and three clear phases (hexane, water and perfluorooctane from top to bottom) are observed after 3 min settling (Figure S13c), further verifying that the perfluorocarbon chain of perfluorooctane is both hydrophobic and oleophobic.

To compare the oleophobicity of perfluorocarbon chain for different oils, different organic solvents with increasing polarity in the order of hexane < toluene < octanol < acetone < acetonitrile are first mixed with perfluorooctane at 150 rpm for 30 min, and then settle for different times. After shaking, the emulsification increases in the order of hexane-perfluorooctane < toluene-perfluorooctane < acetone-perfluorooctane < acetonitrile-perfluorooctane, consistent with the polarity order of these solvents (Figure S14). After 8 min settling, the emulsification of four mixtures all disappears, and the higher polar solvents require longer time for emulsion disappearance. According to the emulsification formation and disappearance order, the oleophobicity of perfluorooctane is dependent on the solvent polarity, i.e., perfluorooctane has stronger oleophobicity for solvents with lower polarity. Different from other organic solvents, octanol forms the emulsion with perfluorooctane in the upper layer. The reason might be attributed to the amphiphilic molecular structure of octanol, and perfluorooctane droplets may enter and disperse into upper octanol phase. The octanol-perfluorooctane mixture has the similar emulsification to the acetone-perfluorooctane mixture, and its emulsion disappears after 3 min, faster than that of acetonitrile-perfluorooctane mixture (8 min). The emulsion should be caused by little liquid droplets rather than air bubbles, since there are not surfactants to stabilize the air bubbles in the mixture. Therefore, the hydro-oleophobic property of perfluorocarbon chain may affect the environmental behavior of PFOS in waters if contaminated by oils, being significantly different from hydrocarbon surfactants.

### Interfacial Distribution Mechanism of PFOS in Oil-Water Mixture

**Figure 8:**
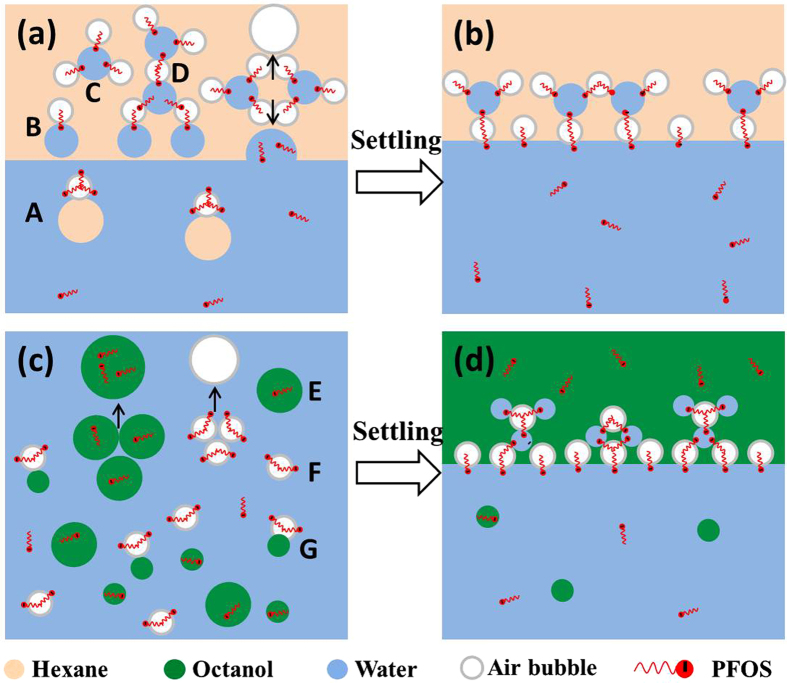
Schematic diagram for PFOS distribution in hexane-water (**a**: after shaking; **b**: after settling) and octanol-water (**c**: after shaking; **d**: after settling) mixtures in the presence of air bubbles.

Based on the aforementioned results and discussion, a schematic diagram for the interfacial distribution of PFOS in hexane-water and octanol-water mixtures is proposed (Fig. 8). The air bubbles are essentially required to form the emulsion with PFOS molecules at the interfaces of air bubbles. The involvement of air bubbles is also verified by the increased volume after shaking in the presence of air for both hexane-water and octanol-water mixtures (Figure S15). The hydrocarbon surfactants such as OS may emulsify oil-water mixture via the stretching of nonpolar hydrocarbon chain into oils (Figure S16), but the strong oleophobicity of perfluorocarbon chain in PFOS prevents the formation of that type of emulsion. The hydro-oleophobic perfluorocarbon chain of PFOS is prone to stretching into air bubbles with sulfonic group in water^29^.

In the hexane-water system, air bubbles may exist in water and hexane phases during the shaking process, and big water and hexane droplets are present in the mixture after shaking although they are not completely mixed due to the hydrophobicity of hexane (Fig. 8a). The air bubbles attached by hexane droplets (model A) in water phase may go up quickly and enter hexane phase, leading to the fast water clarification and the decrease of PFOS concentration in water within 10 min settling. The air bubbles in hexane phase may attach on water droplets with PFOS at the interfaces (models B and C). When more water droplets and air bubbles approach each other, the aggregates like model D may be formed. Because of the strong hydrophobicity of hexane, water droplets should be covered by air bubbles to make them stable in hexane phase. According to the significant decrease of PFOS concentrations in hexane and increase of PFOS concentrations in water in the settling process (Fig. 5), the emulsion is not stable. Water droplets may re-enter into water phase, and small air bubbles could merge into big ones and disappear on hexane surface. After settling, only a few water-air bubble aggregates remain at the bottom of hexane phase, and PFOS concentrations in water increase significantly (Fig. 8b).

In the octanol-water system, the mixture is emulsified well after shaking, and it takes much longer time for water phase to become transparent. Octanol droplets are dispersed in water phase (model E), and they may also attach on air bubbles (model F) to form the aggregates (model G) (Fig. 8c). Besides the dissolution of PFOS in water and octanol, PFOS molecules may also partition to the interfaces of air bubbles and water. Big octanol droplets would quickly coalesce and form the octanol layer after 10 min settling. The slow clarification in water phase may be attributed to the small octanol droplets. Air bubbles may also merge together and release the accumulated PFOS into water and octanol phase. After 2 h settling, PFOS concentrations in both water and octanol almost keep constant, and the aggregates of water droplets and air bubbles exist at the bottom of octanol phase (Fig. 8d). The similar “bridge” effect of PFOS in hexane in Fig. 8a might also exist in the octanol phase, but the possible difference is that more water droplets are attached on one air bubbles, rather than more air bubbles on one water droplet in hexane, due to the higher hydrophilicity of octanol.

## Conclusions

This study clarifies that the hydro-oleophobic perfluorocarbon chain of PFOS repels hydrophobic organic compounds. PFOS can only emulsify oil-water mixture in the presence of air bubbles, different from hydrocarbon surfactant OS, and PFOS accumulation at the surface of air bubbles in hexane and octanol phases causes the significant decrease of PFOS concentrations in water. The emulsion gradually disappears and the concentrated PFOS is released into water during the settling process. Because air bubbles and oils are ubiquitous in surface water or wastewater, this new finding has important implications for the transport and fate of PFOS or other PFCs in aquatic environments, oil-contaminated soil, and the accumulation of PFOS in animal bodies. PFOS accumulation at air bubble surfaces implies the possible approaches for efficient removal of PFOS from water or wastewater. For example, it may be of value for air trapping and flotation treatment processes to remove PFOS from wastewater; enhanced removal of PFOS by adsorption or flocculation may also be achieved with mechanical stirring or air bubbling methods.

## Methods

### Materials

Perfluorooctane, PFOS(K), and OS (all purity > 98%) were purchased from Sigma-Aldrich Co. Methanol, acetone, acetonitrile, toluene, hexane were purchased from Avantor Performance Materials Co. Ethanol, propanol, butanol, hexanol, octanol, carbon tetrachloride were purchased from Beijing Chemical Works.

### PFOS Solubility Experiments

Excess PFOS powder was added into 10 mL of different solvents in 20-mL vials. All the vials were shaken at a speed of 150 rpm at 25 °C for 96 h for the dissolving of the PFOS. After shaking, the vials were settled for 24 h, and 2 mL of the supernatant was taken for PFOS concentration measurements. To avoid the disturbance of organic solvents for PFOS measurement, all samples were dried by a nitrogen evaporation system, and the residues were re-dissolved in 20 mL methanol for PFOS measurement. All experiments were operated thrice and the mean value was adopted.

### Emulsification Experiments

Except for special cases, the initial concentrations of PFOS and OS in aqueous solution were all set at 10 mg/L and 4.3 mg/L, respectively, and the vials were shaken at a speed of 150 rpm at 25 °C for 24 h. To investigate the effect of shaking speed and time, 15 mL of PFOS aqueous solution and 5 mL of hexane were added into 30-mL glass vials, and all vials were shaken at 150 rpm for different period of time or at different rotating speeds for 3 h. The effect of different volumes of air in the system was conducted with hexane/PFOS solution (1/3, v/v) in 60-mL glass vials, and the total volume was changed from 10 to 60 mL. To examine the effect of different hexane volume, 15 mL of PFOS solution and different volume of hexane (1, 2.5, 5, 7.5, 10, and 15 mL) were added into 30-mL vials. To evaluate PFOS distribution in the presence or absence of air, hexane/octanol and PFOS/OS solution (1/3, v/v) were added into 30-mL vials, and the total volume was set at either 20 mL or 32 mL (i.e., the total volume of the vials). For the vials without air, a magneton was added to help mixing. In the investigation of the effect of air at different initial PFOS concentrations, the initial PFOS concentrations in aqueous solution were set at 5, 10, 50, and 100 mg/L. For hexane-water system, photos were taken after shaking, and samples were taken from the top of emulsified hexane layer and the bottom of water layer. For octanol-water system, similar operation was adopted after 10 min settling. All the experiments were operated thrice and the mean value was adopted.

### Emulsion Settling Experiments

To investigate the fate of PFOS in the oil-water mixture, bottle test was conducted to settle for different periods of time and PFOS concentrations in oil and water phases were measured to reflect the stability of emulsion. Initial PFOS concentration in aqueous solution was set at 0.0186 mmol/L. PFOS solution (15 mL) and hexane or octanol (5 mL) were added into 30-mL vials, and the vials were shaken at 150 rpm for 24 h, followed by settling for different periods of time. The top of the organic phase and the bottom of water layer were sampled at the predetermined time for PFOS measurement.

### PFOS and OS Measurement

A nitrogen evaporator was used to remove the organic solvents in the samples, and the residues were dissolved in methanol to reach constant volume. All PFOS and OS concentrations were measured using external standard method, and their calibration curves are shown in Figure S17. High PFOS concentrations (>1 mg/L) and OS concentration were determined by a LC-10ADvp HPLC with a CDD-6A conductivity detector from Shimadzu (Japan). A mixture of methanol/0.02 mol/L NaH_2_PO_4_ solution (70/30, v/v) was selected as the mobile phase, and the limit of detection (LOD) was 0.149 mg/L for PFOS and 0.033 mg/L for OS. Low PFOS concentrations (<1 mg/L) were determined by high performance liquid chromatography-tandem mass spectrometry (HPLC-MS/MS) with an UltiMate 3000 HPLC (Dionex by Thermo Fisher Scientific Inc., MA, USA) equipped with an API 3200 triple quadrupole mass spectrometer (AB SCIEX, ON, Canada). The mobile phase consisted of a binary mixture of 10 mmol/L ammonium acetate and methanol with a total flow rate of 0.3 mL/min. The LOD was measured to be 0.112 μg/L.

## Additional Information

**How to cite this article**: Meng, P. *et al*. Effect of hydro-oleophobic perfluorocarbon chain on interfacial behavior and mechanism of perfluorooctane sulfonate in oil-water mixture. *Sci. Rep.* **7**, 44694; doi: 10.1038/srep44694 (2017).

**Publisher's note:** Springer Nature remains neutral with regard to jurisdictional claims in published maps and institutional affiliations.

## Supplementary Material

Supporting Information
