# Supplementary material for: Effect of hydro-oleophobic perfluorocarbon chain on interfacial behavior and mechanism of perfluorooctane sulfonate in oil-water mixture
Source: Sci Rep. 2017 Mar 16;7:44694. doi: 10.1038/srep44694 (PMC5353549; doi:10.1038/srep44694)
Supplement: Supporting Information [file srep44694-s1.pdf]

Supplementary information for:

Effect of hydro-oleophobic perfluorocarbon chain on interfacial behavior and mechanism of perfluorooctane sulfonate in oil-water mixture

Pingping Meng [1], Shubo Deng [1,*], Ziwen Du [1], Bin Wang [1, *], Jun Huang [1], Yujue Wang [1], Gang Yu [1], Baoshan Xing [2]

[1] State Key Joint Laboratory of Environment Simulation and Pollution Control, School of Environment, POPs Research Center, Tsinghua University, Beijing, 100084, China

[2] Stockbridge School of Agriculture, University of Massachusetts, Amherst, Massachusetts, 01003, USA

[*] Corresponding author, Tel.: +86-10-62792165. Fax: +86-10-62794006.

E-mail: dengshubo@tsinghua.edu.cn (S. Deng); thuwb@tsinghua.edu.cn (B. Wang)

This file includes 10 pages, 17 figures on:

- ◆Photos for the mixture of hexane and PFOS solution at different shaking times with shaking speed of 150 rpm (Figure S1)
- ◆Photos for the mixture of hexane and PFOS solution at different shaking speed for 3 h (Figure S2)
- ◆Photos for the effect of mixture volume on PFOS distribution after shaking the hexane-water (1:3 v/v) mixture at 150 rpm for 24 h in 60-mL vials (Figure S3)
- ◆Effect of mixture volume on PFOS distribution after magnetic stirring the hexane-water (1:3 v/v) mixture at 500 rpm for 3 h in 50-mL conical flasks (the initial PFOS concentration in water was 10 mg/L, and the total volume of flask is about 58 mL) (Figure S4)
- ◆Effect of hexane volume on PFOS concentrations in water after shaking the hexane-water mixture (15 mL of PFOS solution) at 150 rpm for 24 h (Figure S5)
- ◆Water droplets in the hexane observed via a microscope (Leica, DM6000B, Germany) (Figure S6)
- ◆Size distribution of the hexane-water emulsion using a laser particle size analyzer (Beckman Coulter, LS13320, USA) (Figure S7)
- ◆Effect of initial PFOS concentrations on PFOS distribution in hexane-water mixture after 0 min settling and octanol-water mixture at 10 min settling after shaking at 150 rpm for 24 h in the presence or absence of air (Figure S8)
- ◆PFOS concentrations in water during the settling process after 15 mL of 10.5 mg/L PFOS solution in 30-mL vial for shaking at 150 rpm for 3 h (Figure S9)
- ◆Photos for PFOS solution at different settling times after shaking at 150 rpm for 3 h (Figure S10)
- ◆Effect of initial PFOS concentrations on its distribution in water in the hexane-water mixture at different settling times after shaking at 150 rpm for 3 h (Figure S11)

◆Photos for the hexane-water mixture with different PFOS concentrations at different settling times after shaking at 150 rpm for 3 h (Figure S12)

◆Stratification of perfluoroctane with water, hexane or both of them in 30-mL glass tubes for 3 min settling (Figure S13)

◆Stratification of perfluoroctane and different organic solvents after shaking and then settling for different times (Figure S14)

◆Volume change of hexane-water and octanol-water mixtures after shaking at 150 rpm and 25℃ for 24 h. (Figure S15)

◆Schematic diagram for emulsion formation with the help of OS in the mixture of hexane/octanol-water (Figure S16)

◆The calibration curves for PFOS and OS. (a) PFOS calibration curves, PFOS concentration < 1 mg/L, (b) PFOS calibration curves, PFOS concentration > 1 mg/L, (c) PFOS calibration curves. (Figure S17)

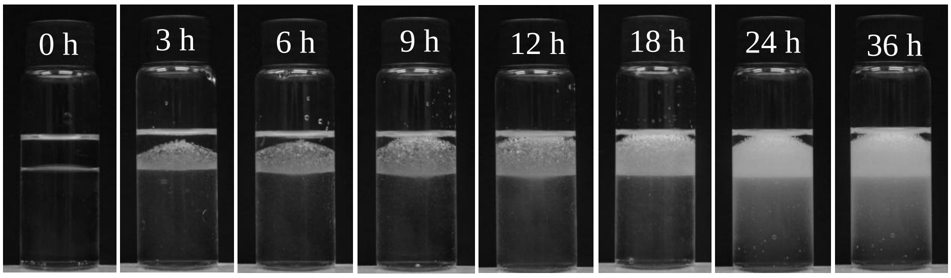

Figure S1. Photos for the mixture of hexane and PFOS solution at different shaking times with shaking speed of 150 rpm

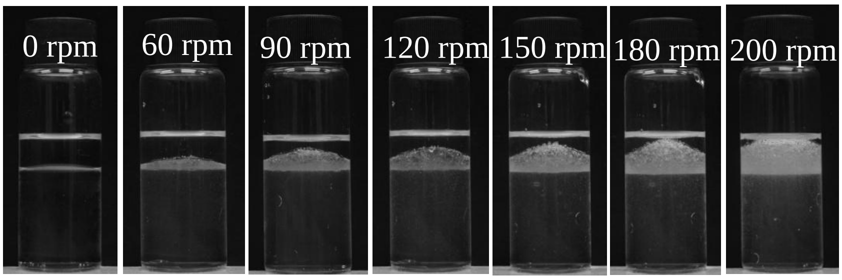

Figure S2. Photos for the mixture of hexane and PFOS solution at different shaking speed for 3 h

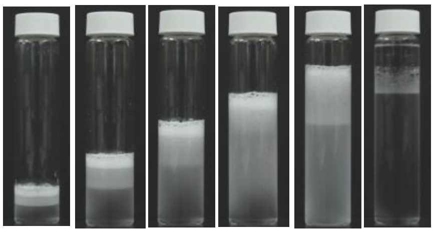

Figure S3. Photos for the effect of mixture volume on PFOS distribution after shaking the hexane-water (1:3 v/v) mixture at 150 rpm for 24 h in 60-mL vials

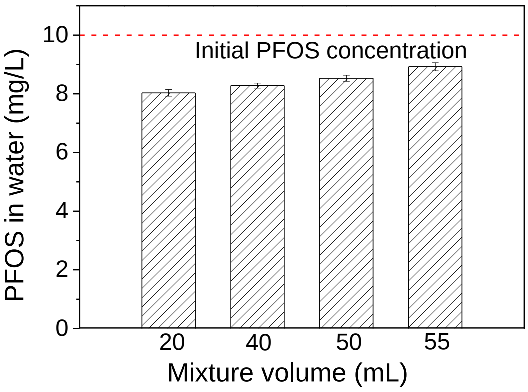

Figure S4. Effect of mixture volume on PFOS distribution after magnetic stirring the hexane-water (1:3 v/v) mixture at 500 rpm for 3 h in 50-mL conical flasks (the initial PFOS concentration in water was 10 mg/L, and the total volume of flask is about 58 mL)

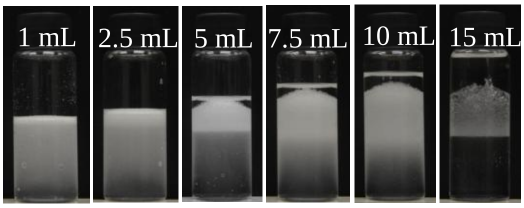

Figure S5. Effect of hexane volume on PFOS concentrations in water after shaking the hexane-water mixture (15 mL of PFOS solution) at 150 rpm for 24 h

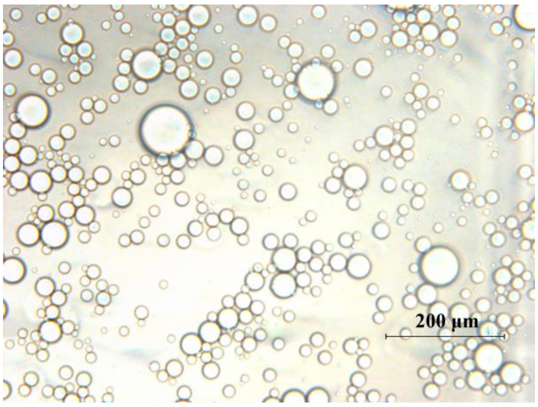

Figure S6. Water droplets in the hexane observed via a microscope (Leica, DM6000B, Germany)

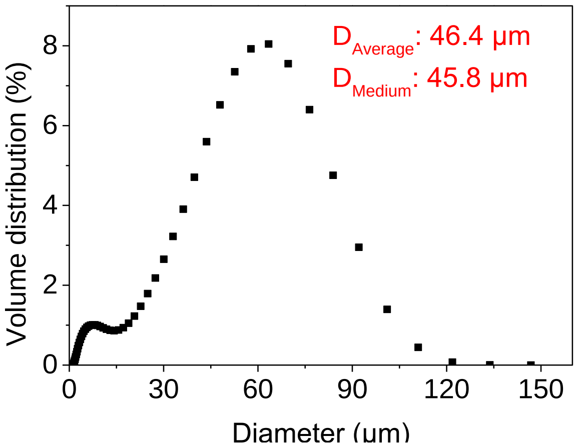

Figure S7. Size distribution of the hexane-water emulsion using a laser particle size analyzer (Beckman Coulter, LS13320, USA)

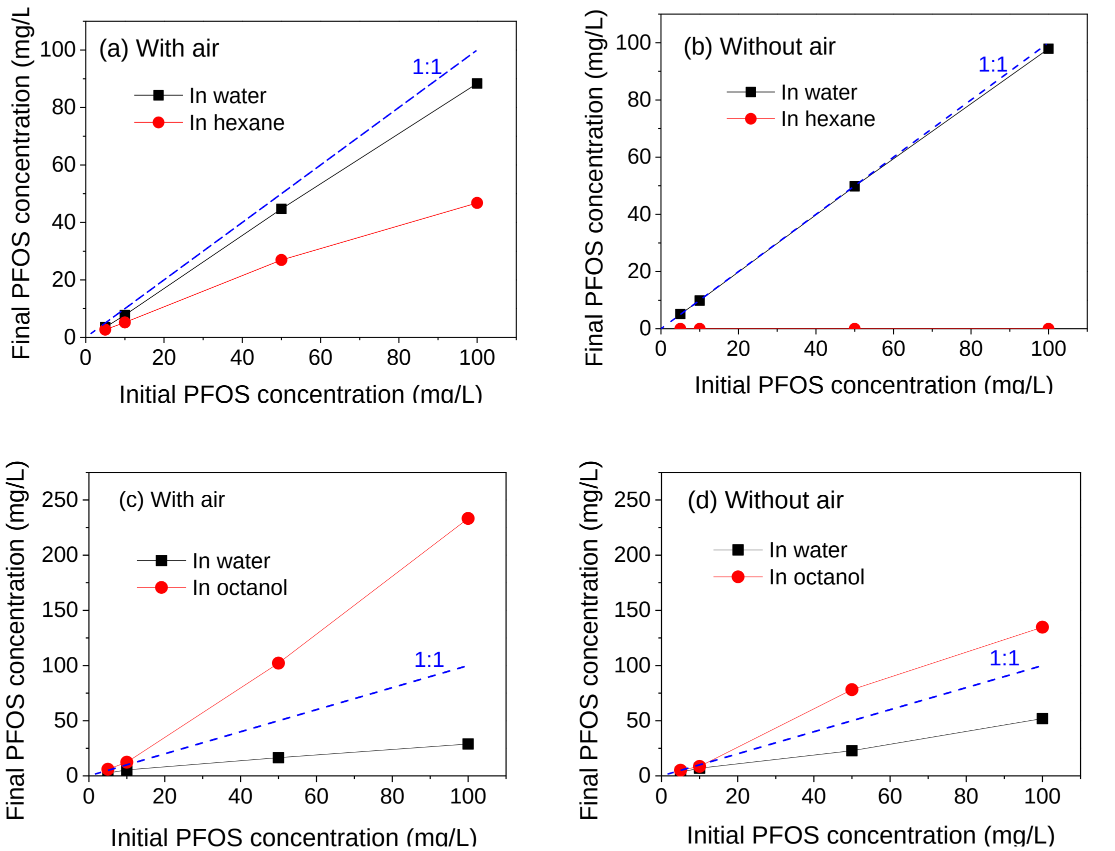

Figure S8. Effect of initial PFOS concentrations on PFOS distribution in hexane-water mixture after 0 min settling and octanol-water mixture at 10 min settling after shaking at 150 rpm for 24 h in the presence or absence of air

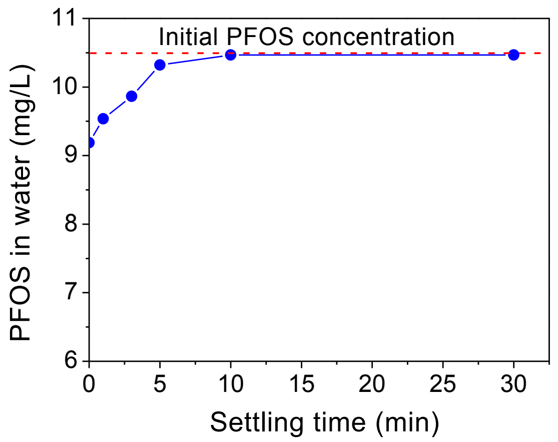

Figure S9. PFOS concentrations in water during the settling process after 15 mL of 10.5 mg/L PFOS solution in 30-mL vial for shaking at 150 rpm for 3 h

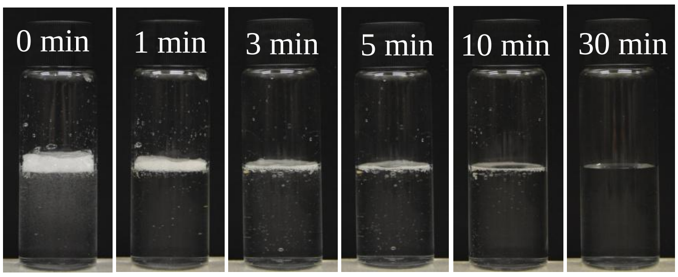

Figure S10. Pictures for PFOS solution at different settling time after shaking at 150 rpm for 3 h

Emulsion Settling Experiments. In the investigation of the effect of initial PFOS concentrations, 30 mL of PFOS solution and 10 mL of hexane were added into 50-mL polyprolene (PP) centrifuge tubes, and the initial PFOS concentrations were from 0.0186 μmol/L to 0.00186 mmol/L. All the pipes were shaken at a speed of 150 rpm at 25℃ for 3 h. The vials/tubes were taken out and put on a table for settling, and the samples from the top of the organic phase and the bottom of water layer were taken at the predetermined time for PFOS measurement. For the experiments conducted in PP tubes, solution was transferred into 60-mL glass vials before taking photos.

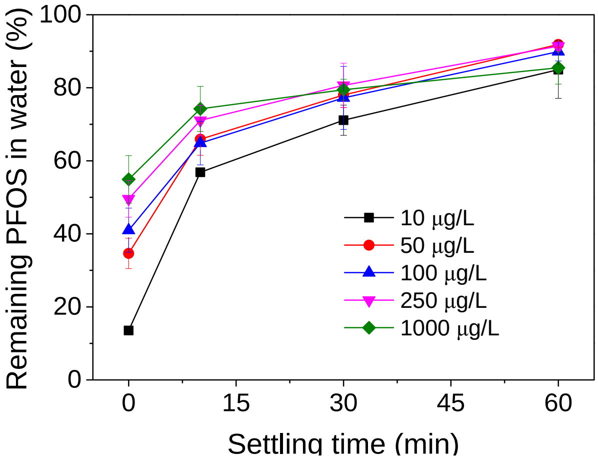

Figure S11. Effect of initial PFOS concentrations on its distribution in water in the hexane-water mixture at different settling time after shaking at 150 rpm for 3 h

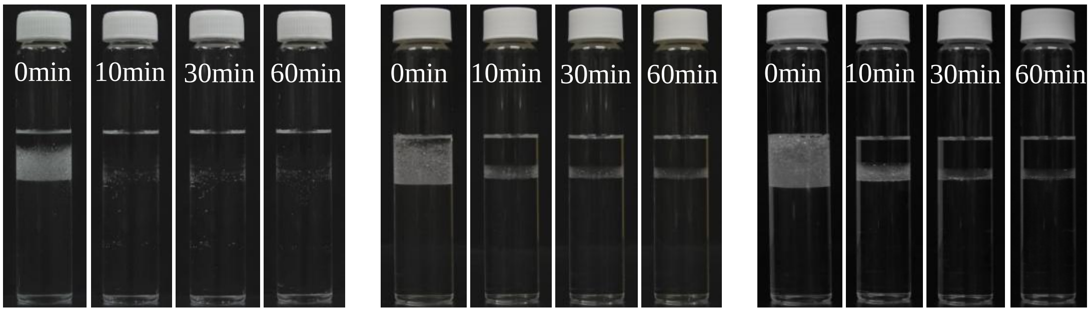

(a) 10 μg/L (b) 50μg/L (c) 100 μg/L

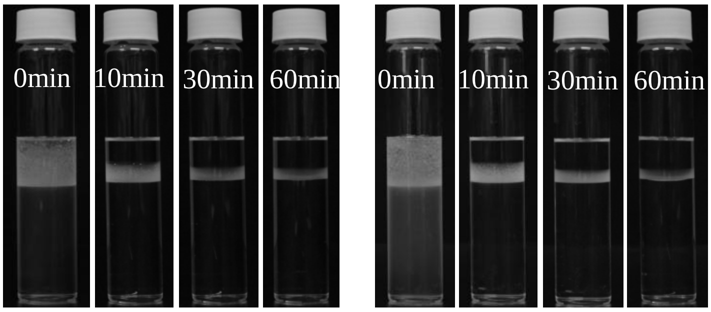

(d) 250 μg/L (e) 1000 μg/L

Figure S12. Photos for the hexane-water mixture with different PFOS concentrations at different settling times after shaking at 150 rpm for 3 h

Solvent Stratification Experiments. Twenty milliliters of perfluoroctane/different solvents (water, hexane, toluene, carbon tetrachloride, acetone, acetonitrile and octanol) (1/1, v/v) or 20 mL of perfluoroctane/water/hexane (1/1/1, v/v/v) were added into 30-mL glass vials. All vials were shaken in a shaker at a speed of 150 rpm at 25℃ for 30 min. Finally, all vials were taken out and photos were taken at different times.

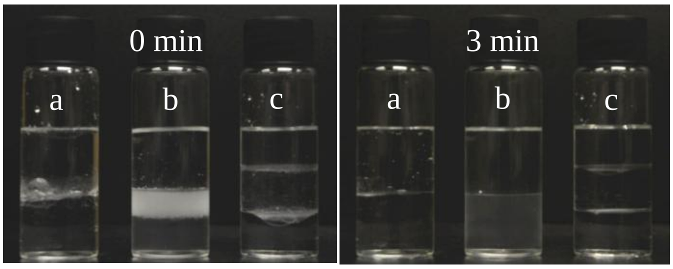

Figure S13. Stratification of perfluoroctane with water (a), hexane (b) or both of them (c) in 30-mL glass tubes for 3 min settling

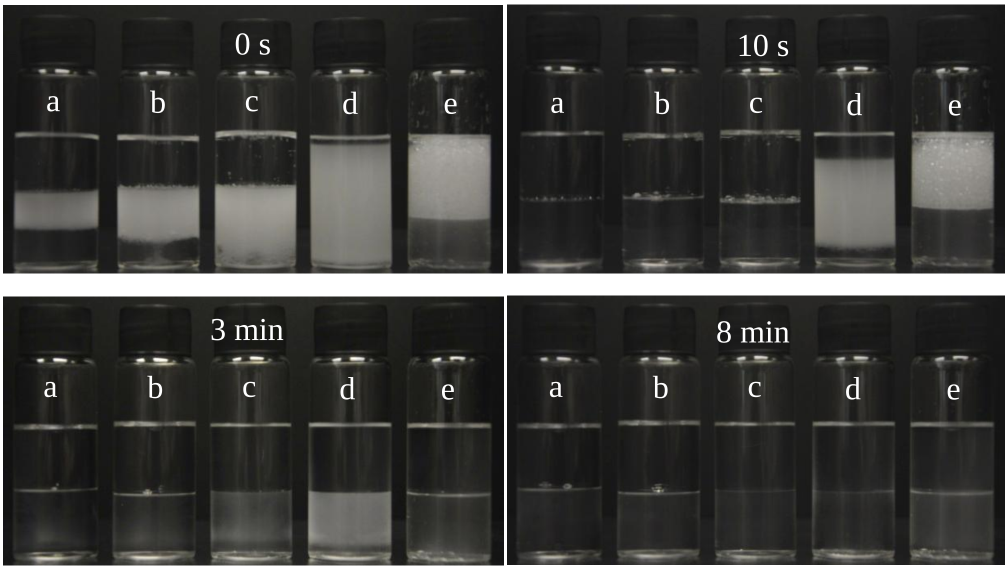

Figure S14. Stratification of hexane-perfluoroctane (a) toluene-perfluoroctane (b), acetone-perfluoroctane (c), acetonitrile-perfluoroctane (d), and octanol-perfluoroctane (e) mixtures after shaking and then settling for different times

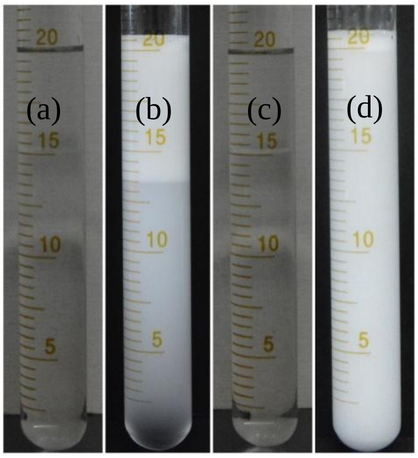

Figure S15. Volume change of hexane-water and octanol-water mixtures after shaking at 150 rpm and 25℃ for 24 h. (a) 5 mL of hexane in 15 mL water system after shaking (b) 5 mL of hexane in 15 mL of PFOS solution before shaking, (c) 5 mL of octanol in 15 mL of water after shaking, and (d) 5 mL of octanol in 15 mL of PFOS solution before shaking

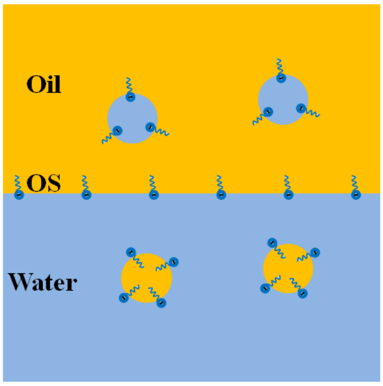

Figure S16. Schematic diagram for emulsion formation with the help of OS in the mixture of hexane/octanol-water

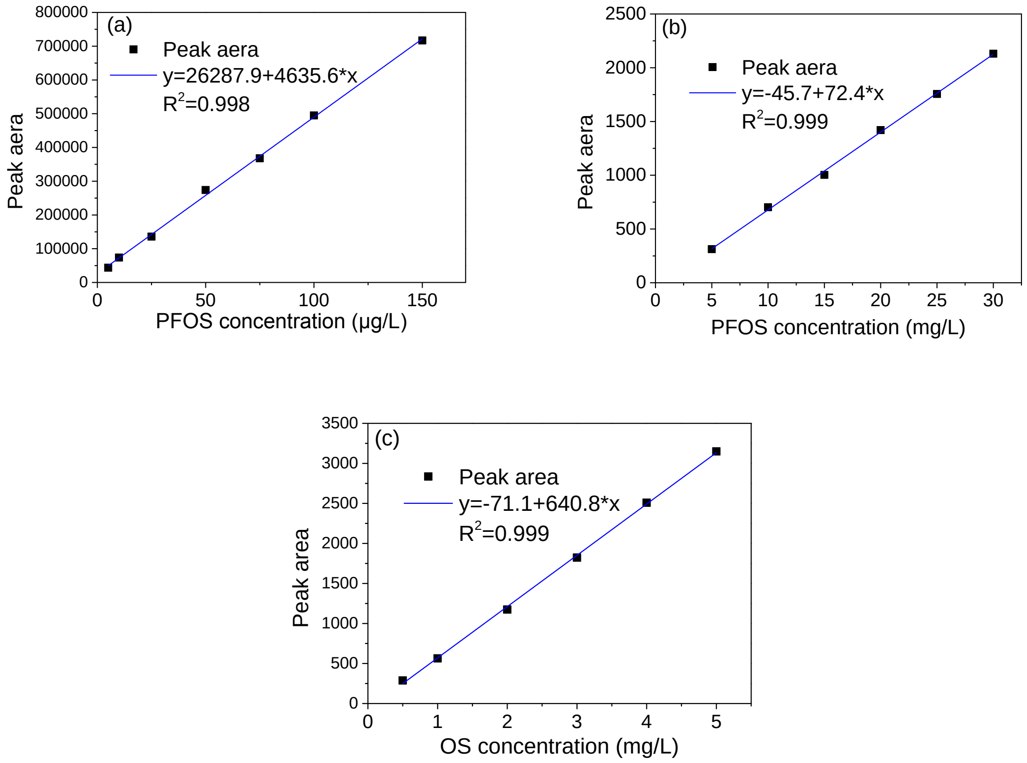

Figure S17. The calibration curves for PFOS and OS. (a) PFOS calibration curves, PFOS concentration < 1 mg/L, (b) PFOS calibration curves, PFOS concentration > 1 mg/L, (c) PFOS calibration curves
